# Transparent Development of the WHO Rapid Advice Guidelines

**DOI:** 10.1371/journal.pmed.0040119

**Published:** 2007-05-29

**Authors:** Holger J Schünemann, Suzanne R Hill, Meetali Kakad, Gunn E Vist, Richard Bellamy, Lauren Stockman, Torbjørn Fosen Wisløff, Chris Del Mar, Frederick Hayden, Timothy M Uyeki, Jeremy Farrar, Yazdan Yazdanpanah, Howard Zucker, John Beigel, Tawee Chotpitayasunondh, Tran Tinh Hien, Bülent Özbay, Norio Sugaya, Andrew D Oxman

## Abstract

Emerging health problems require rapid advice. We describe the development and pilot testing of a systematic, transparent approach used by the World Health Organization (WHO) to develop rapid advice guidelines in response to requests from member states confronted with uncertainty about the pharmacological management of avian influenza A (H5N1) virus infection. We first searched for systematic reviews of randomized trials of treatment and prevention of seasonal influenza and for non-trial evidence on H5N1 infection, including case reports and animal and in vitro studies. A panel of clinical experts, clinicians with experience in treating patients with H5N1, influenza researchers, and methodologists was convened for a two-day meeting. Panel members reviewed the evidence prior to the meeting and agreed on the process. It took one month to put together a team to prepare the evidence profiles (i.e., summaries of the evidence on important clinical and policy questions), and it took the team only five weeks to prepare and revise the evidence profiles and to prepare draft guidelines prior to the panel meeting. A draft manuscript for publication was prepared within 10 days following the panel meeting. Strengths of the process include its transparency and the short amount of time used to prepare these WHO guidelines. The process could be improved by shortening the time required to commission evidence profiles. Further development is needed to facilitate stakeholder involvement, and evaluate and ensure the guideline's usefulness.

## Abstract 2

The authors describe the development and pilot testing of a systematic, transparent approach to develop rapid advice guidelines on pharmacological management of avian influenza.

Clinical practice guidelines generally, and some WHO guidelines specifically, have been criticized for not being based on the best available evidence, for being exposed to undue influence by industry and experts who participate in guideline panels, and for not adhering to guidelines for preparing guidelines [1–7]. Guidance that is not informed by the best available evidence or by statements that the available evidence is of low quality can harm patients, waste limited resources, and hinder research to address important uncertainties [8].

While there is broad agreement that rigorous and transparent methods should be used [9–12], rigorous development of guidelines can take two years or more [13,14]. This timeframe is not practical for providing rapid advice, for example for emerging infectious diseases such as avian influenza (H5N1 infection) or severe acute respiratory syndrome (SARS). Indeed, one of the most frequently cited weaknesses in guideline development is the length of time that it takes to develop a guideline [15]. Organizations including the National Centre for Health and Clinical Excellence in the United Kingdom and the National Institutes of Health in the United States are investigating ways of streamlining guidelines development processes [16,17].

The WHO Advisory Committee for Human Research, an independent committee appointed by the Director-General of the WHO, evaluated existing WHO and other guidance processes and suggested ways to improve WHO's methods[18,19]. Consistent with these recommendations, a new model for developing WHO rapid advice guidelines was designed and tested through the development of guidelines for the pharmacological management of avian influenza A (H5N1) virus infection [20,21]. Because the approach to developing these rapid advice guidelines was novel for WHO, we describe the methods, the strengths of the approach, and ways in which this approach should be further developed.

## The Process

In January 2006, the WHO decided to convene a rapid advice guidelines panel for the pharmacological management of H5N1 patients in response to requests for advice from frontline clinicians and public health professionals managing H5N1 infections. The key steps and timeline for developing the guidelines are summarized in Box 1.

**Box 1. Key Steps in the Development of WHO Rapid Advice Guidelines**

**Table:**
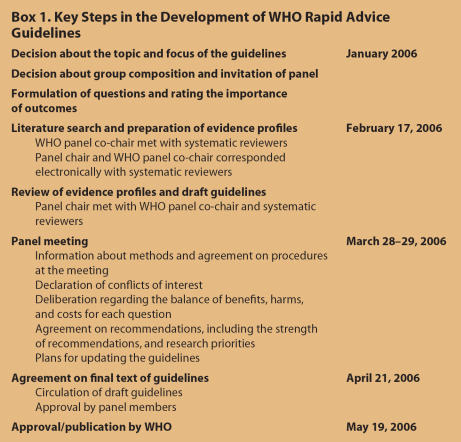

### Group selection and composition

In selecting members for the panel, we wanted to include several important stakeholders: clinical, methodological, and basic science experts and member country representatives, including low- and middle-income countries. We used the WHO's international network of response teams for viral pandemics and searched the medical literature for experts on H5N1 infection to identify panel members. We asked methodologists with experience in applying the Grading Recommendations Assessment, Development and Evaluation (GRADE) approach that was officially adopted by WHO to participate in the creation of evidence profiles and the guideline development process. Thirteen voting panel members supplemented by WHO experts participated in the panel meeting. We followed a thorough process to declare conflicts of interest (see Text S1 under Supporting Information).

### Formulating questions and rating the importance of outcomes

**Figure 1:**
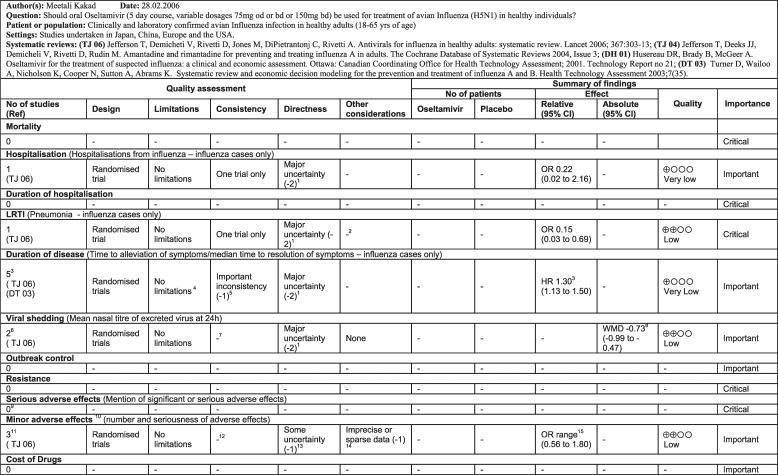
Example of a GRADE Evidence Profile Footnotes 1–15 in [21] provided detailed information about the rationale underlying the decisions.

The original questions were identified by clinicians managing patients with H5N1 infections and refined by panel members. For each question an evidence profile was prepared using the GRADE approach (Figure 1) [22]. The GRADE approach required the identification of relevant outcomes to be included in each evidence profile before developing the evidence profiles. Two reviewers identified potentially important outcomes and this list was circulated to the panel chair, WHO staff, and the scientific reviewers by email for independent scoring of the relative importance of each outcome and identification of additional potentially important outcomes. Outcomes were rated on a scale from 1–9; a rating of 7–9 indicated that the outcome was critical for a decision or recommendation, 4–6 indicated it was important, and 1–3 indicated it was not important. The evidence profiles included only critical or important outcomes based on the mean value of the ratings by the panel members.

To obtain consumer input, the Cochrane Consumer Network was also invited to provide feedback through their electronic discussion list. We received only four responses, but despite reported difficulties with the rating, the relative importance of the outcomes did not differ importantly from those of the panel and no additional outcomes were identified.

### Preparation of evidence profiles

An independent review team searched for systematic reviews and recent randomized trials (published in 2005 or 2006) for the treatment and chemoprophylaxis of any influenza virus infection; and case series, animal studies, and in vitro studies for the treatment or chemoprophylaxis of H5N1 infection (from 1966). The team prepared evidence profiles using the GRADE profiler software (v1.12, http://www.gradeworkinggroup.org) and proposed quality ratings according to the criteria in Box 2. The quality of outcomes measured in each animal study was judged based on whether (1) the pathogenicity of H5N1 virus was tested in the model (e.g., mortality), (2) statistical methods were adequate, and (3) a significant effect was demonstrated.

**Box 2. Key Quality Criteria**

**The quality of evidence for each important or critical outcome is based on:**

- the study design
- limitations of the studies (execution)
- consistency of the evidence across studies
- the directness (generalizability) of the evidence to the population, intervention, comparison, and outcomes
- the precision of the estimate or sparseness of data

**Evidence was classified as “high”, “moderate”, “low”, or “very low” based on these criteria and the following definitions:**

- High: Further research is very unlikely to change confidence in the estimate of effect.
- Moderate: Further research is likely to have an important impact on confidence in the estimate of effect and may change the estimate.
- Low: Further research is very likely to have an important impact on confidence in the estimate of effect and is likely to change the estimate.
- Very low: Any estimate of effect is very uncertain.

**Figure 2:**
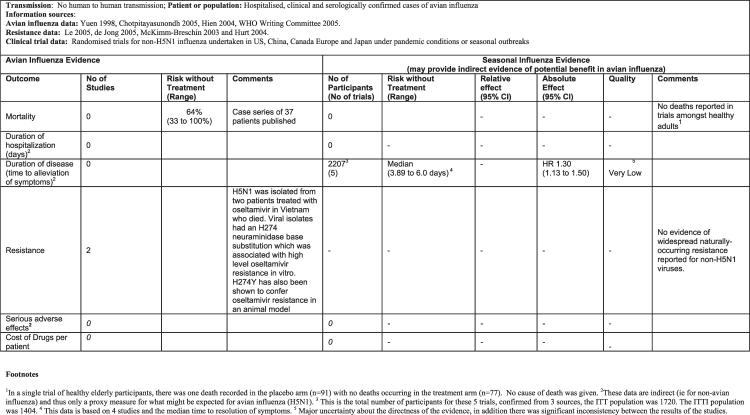
Summary of Findings for the Following Scenario: Should Oseltamivir Be Used for Treatment of Patients Hospitalized with Avian Influenza (H5N1)?

A summary of the findings for each question, including both trial evidence for non-H5N1 influenza and the available evidence for H5N1 infection, was prepared for each question (Figure 2) [21].

### Panel meeting

Guideline group members and an independent expert received evidence profiles about two weeks prior to the meeting, including information about the applied methods. They were asked to identify any important missing evidence.

A draft of the guidelines was prepared by the WHO secretariat and the panel chair prior to the meeting. For the actual meeting, the agreed process rules were that: (1) Additional evidence would only be allowed at the meeting if it had been omitted from the evidence summaries, or was new and critical for decision making; (2) The GRADE approach would be used to grade the quality of evidence and the strength of recommendations [10,21]; (3) Recommendations would be based on a consensus of the panel and voting would be used if agreement could not be reached; (4) All panel members would be asked to consider their own and other conflicts during the discussion and decision making and to abstain from discussion and voting if necessary (see Supporting Information); (5) Subsequent interaction and discussion would take place through email but recommendations would not be changed after the meeting, except for minor wording changes or correction of factual errors.

### Deliberation regarding the balance of benefits, harms, and costs

For each intervention considered, the panel formulated a recommendation based on the panel members' judgments regarding the balance between the benefits, harms (adverse effects), burdens (e.g., taking medication daily), costs, and values and preferences (the desirability or preference that individuals exhibit for a particular outcome) of the intervention (see Supporting Information Text S1 for information on cost).

Recommendations were classified as “strong” or “weak.” The panel was informed that strong recommendations should be interpreted as: (1) Most individuals should receive the intervention; (2) Most well-informed individuals would want the recommended course of action and only a small proportion would not; (3) The intervention could unequivocally be used in policy making.

Weak recommendations were to be interpreted as: (1) The majority of well-informed individuals would want the suggested course of action, but an appreciable proportion would not; (2) Values and preferences related to this intervention are likely to vary widely; (3) Policy making will require extensive debates and involvement of many stakeholders.

**Figure 3:**
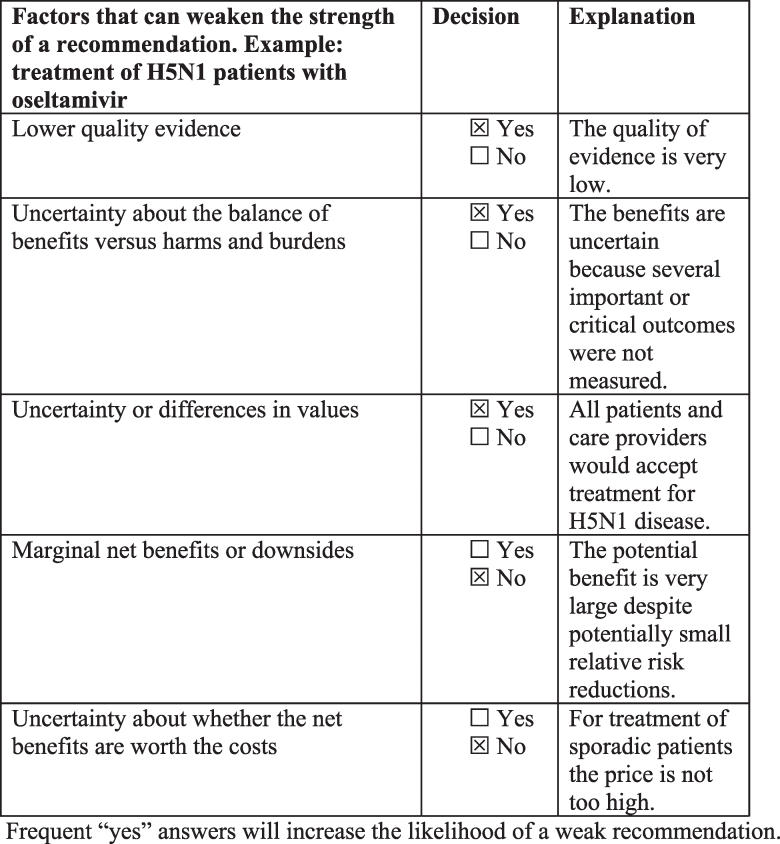
Decisions about the Strength of a Recommendation

The panel used the factors listed in Figure 3 as a basis for going from a strong to a weak recommendation. This information was recorded for recommendations where a formal vote was required, as illustrated in Figure 3.

## Outcomes

It took approximately one month to put together a team to prepare the evidence profiles, but once the team was assembled it took only five weeks to prepare and revise the evidence profiles and prepare draft guidelines prior to the panel meeting (Box 1). A draft manuscript for publication was prepared within 10 days following the panel meeting. It took only two additional weeks to complete a final draft of the document (on April 21, 2006) that included all of the considered evidence and recommendations.

Overall the quality of the underlying evidence for all recommendations was very low because the evidence was based upon observational data from case series describing small numbers of patients infected with avian influenza A (H5N1) virus, laboratory research, or on extrapolation from randomized trials of treatment and prophylaxis for seasonal influenza. The panel evaluated the rationale for the quality rating during the meeting and subsequently via electronic correspondence. The reasons for considering the evidence to be of low or very low quality (i.e., for having little confidence in the available estimates of effect) were: (1) the lack of direct evidence from trials among patients with H5N1 infection or exposure, (2) lack of evidence for important outcomes for H5N1 infection that were not common or not measured in seasonal influenza trials, and (3) sparse data for other important outcomes. Additional factors and specific judgments regarding the quality of the evidence are included in detailed footnotes in the evidence profiles. The panel expressed concern about the categories used to grade the quality of evidence. For example, although the quality of the evidence for treatment and chemoprophylaxis with oseltamivir and zanamivir was very low for both, there was nonetheless a difference in the quality of the evidence within the categories for these two drugs.

The panel considered several different specific patient and exposure groups when considering the balance between benefits, harms, and costs for chemoprophylaxis. This led them to develop a risk categorization for exposure to assist decision makers in prioritizing use of antivirals [20,21].

Of the 27 recommendations, 15 were strong recommendations, but most (11) were strong recommendations against a specific intervention. The arguments for making strong recommendations for specific interventions, despite the low quality of the available evidence, were the high risk of serious outcomes including death, the lack of alternative treatments, a low risk of serious adverse effects based on the available evidence, relatively low costs, and a possibility of beneficial effects, although there is much uncertainty about these. The strong recommendation regarding treatment of H5N1 patients with oseltamivir required a vote (one panel member abstained and one voted for a weak recommendation). The main argument that led to voting about this recommendation was that it is uncertain whether the intervention does more good than harm in the face of very low-quality evidence. Only one other recommendation required voting. Other factors that influenced the recommendations, value judgments, and other directly relevant information are described in the remark sections following each recommendation [21].

## Discussion

These guidelines were prepared in less than two months through an intense international collaboration, the use of electronic communication, and one panel meeting. However, it took one additional month initially to put together the team that prepared the evidence profiles.

There are at least two ways in which the time needed to prepare rapid advice could be shortened: first, by identifying or establishing collaborating centres with the competency needed to prepare evidence profiles and second, by building up in-house capacity to reduce the time needed to organize a review team.

### Strengths of the process

Strengths of the applied process include its transparency and the short amount of time used to prepare the guidelines. Guideline developers are increasingly using the GRADE approach because it includes transparent judgments about each of the key factors that determine the quality of evidence for each important outcome, and overall across outcomes for each recommendation [10,22,23]. In addition, the approach used in developing these guidelines included transparent consideration of the key factors that determine the strength of a recommendation. The rapid preparation of evidence profiles was possible because of the availability of high-quality systematic reviews of the evidence from seasonal influenza and the involvement of a review team with experience in preparing evidence profiles and relevant clinical expertise. In the absence of higher-quality direct evidence, the panel considered case reports, animal studies, and in vitro studies for H5N1, as well as the available indirect evidence from systematic reviews of clinical trials for seasonal influenza, systematically and transparently.

The broad representation of stakeholders in the guideline group allowed the inclusion of different perspectives for making informed judgments about the importance of outcomes, the quality of evidence, and the strength of recommendations. The publicly available evidence profiles facilitate adaptation of the guidelines to specific settings and updating of the guidelines, as well as contributing to transparency [13,21,24].

### Limitations of the process

Limitations of this process relate to its very purpose: providing rapid advice. Thus, the time available for developing these guidelines did not permit detailed consideration of all clinical questions that clinicians may face. For example, the discussions about whether to use prophylactic antibiotics and which recommendations apply to situations of human-to-human transmission were short and, therefore, did not result in specific recommendations. It is unlikely that important evidence that would have led to different recommendations was missed given the nature of the problem; i.e., an emerging disease. In areas with an ample evidentiary base, evidence could be missed by relying on systematic reviews of the indirect evidence. However, this generally should not be the case when rapid advice is needed. It is not clear whether the differences the panel identified within the category of very low-quality evidence are truly important for decision making, but panel members requested that this information should not be lost in translating the quality of evidence into the four categories based on the GRADE approach.

The total budget required for this guideline development project amounted to approximately US$150,000, for a focused guideline. This was made possible in part by limiting the number of meetings through the use of electronic communication tools. While this cost compares favourably with the cost of other guideline development processes, it remains out of reach for many countries trying to develop national rapid advice guidelines.

Involvement of stakeholders through consultation is limited by the rapid process. There are at least three ways in which stakeholder involvement could be improved. First, rapid consultations could be facilitated by establishing stakeholder groups and mechanisms for involvement such as those used by the National Centre for Health and Clinical Excellence. Second, evaluation and updating of the guidelines offer opportunities for more stakeholder involvement in revisions of the guidelines. Third, because the guidelines need to be adapted to specific settings, stakeholders could be involved in local adaptation processes.

Although research needs were identified by the guideline panel, these do not provide clear guidance for what research should be prioritized to address the most important uncertainties about pharmacological management of H5N1 infection. While this is partly due to the mandate that was given to the panel, recommendations for research should be viewed as an integral part of making recommendations.

## Conclusion

In summary, we found that it is feasible to develop evidence-based guidelines systematically and transparently in as little as two months. The cost of doing this is prohibitively high for low- and middle-income countries and it would be wasteful for high-income countries to duplicate this process unnecessarily. WHO, or others developing rapid advice, can therefore provide an important service by using a robust and transparent process that simplifies adaptation to specific settings. Further work is required to develop systematic processes for WHO to give even faster or immediate guidance for emerging infectious diseases.

## Supporting Information

Text S1 Information on cost and resource utilization, and declaration and handling of conflicts of interest (25 KB DOC).

Alternative Language Abstract S1 Translation of abstract into German by H. Schünemann (27 KB DOC).

Alternative Language Abstract S2 Translation of abstract into Turkish by B. Özbay (27 KB DOC).

Alternative Language Abstract S3 Translation of abstract into Norwegian by G. Vist (26 KB DOC).

Alternative Language Abstract S4 Translation of abstract into Italian by I. Terrenato, H. Schünemann, and P. Muti (27 KB DOC).

Alternative Language Abstract S5 Translation of abstract into French by Y. Yazdanpanah (27 KB DOC).

Alternative Language Abstract S6 Translation of abstract into Chinese by X. Xu (26 KB DOC).

Alternative Language Abstract S7 Translation of abstract into Pilipino (Tagalog) by E. Dimaano (28 KB DOC).

Alternative Language Abstract S8 Translation of abstract into Portuguese by M. Crusat (26 KB DOC).

Alternative Language Abstract S9 Translation of abstract into Spanish by E. Martinez (27 KB DOC).

Alternative Language Abstract S10 Translation of abstract into Indonesian by T. Aditama (30 KB DOC).

Alternative Language Abstract S11 Translation of abstract into Laos by M. Mayxay (60 KB DOC).

Alternative Language Abstract S12 Translation of abstract into Vietnamese by T. Chau and T. Tuan (34 KB DOC).

Alternative Language Abstract S13 Translation of abstract into Finnish by H. Peltola (26 KB DOC).

Alternate Language Abstract S14 Translation of abstract into Japanese by R. Ochiai (76 KB PDF).

Alternate Language Abstract S15 Translation of abstract into Korean by M. Oh (112 KB PDF).

Alternate Language Abstract S16 Translation of abstract into Thai by S. Chaiyara (117 KB PDF).

Alternate Language Abstract S17 Translation of abstract into Devanagari by A. Arjyal (42 KB PDF).

## Abbreviations

GRADE: Grading Recommendations Assessment, Development and Evaluation
WHO: World Health Organization
