# Supplementary material for: Transparent Development of the WHO Rapid Advice Guidelines
Source: PLoS Med. 2007 May 29;4(5):e119. doi: 10.1371/journal.pmed.0040119 (PMC1877972; doi:10.1371/journal.pmed.0040119)
Supplement: Text S1 — (25 KB DOC). [file pmed.0040119.sd001.doc]

**Supporting information**

**Information on cost and resource utilization**

Resource utilization and cost data were lacking for these guidelines. The review team considered formal economic modelling to be of limited value, given unavailability of reliable estimates for nearly all of the important outcomes as well as resource utilization and cost data. Cost was, however, identified as and important factor in formulating recommendations, particularly for prophylaxis, and recommendations include statements about the values the panel placed on resource utilization.

**Declaration and handling conflict of interest**

All panel members completed written conflict of interest declarations prior to the meeting according to WHO rules. At the beginning of the two day meeting all voting panel members had to again verbally declare conflicts of interest before discussion about the guideline content. Conflicts of interest were described as: direct financial (e.g. stock options), indirect financial (e.g. family members working for the pharmaceutical industry) and personal (e.g. academic career enhancing support received, such as research grants). All panel members were asked to consider their own and other conflicts during the discussion and decision making as well as to abstain from discussion and voting if necessary.
