# Supplementary material for: Transparent Development of the WHO Rapid Advice Guidelines
Source: PLoS Med. 2007 May 29;4(5):e119. doi: 10.1371/journal.pmed.0040119 (PMC1877972; doi:10.1371/journal.pmed.0040119)
Supplement: Alternative Language Abstract S1 — (27 KB DOC). [file pmed.0040119.sd002.doc]

**Translation of the abstract into German by Holger J. Schünemann:**

**Zusammenfassung des Artikels**

**Einführung:** Neu auftretende Krankheiten erfordern schnelles Ratgeben und Handeln. Die Autoren beschreiben die Entwicklung und eine Pilotstudie eines systematischen und transparenten Ansatzes, den die Weltgesundheitsorganisation (WHO) als Antwort auf Anforderungen ihrer Mitgliedsstaaten, die sich im Unklaren befanden, welche pharmakologischen Interventionen sie gegen die Vogelgrippe Influenza A(H5N1) Virus sie anwenden sollten, angewendet hat.

**Methodik:** Methodologen entwickelten Evidenzprofile, die die Ergebnisse von randomisierten und kontrollierten Studien aus systematischen Übersichtsarbeiten zur Behandlung und Prävention der gewöhnlichen Grippe und die Ergebnisse von nicht-randomisierten Studien, inklusive von Falldarstellungen, in vitro und anderen Forschungsarbeiten darstellten. Eine Kommission von klinischen Experten, die H5N1 Patienten behandelten, Influenzawissenschaftlern und Methodologen kamen zu einem zwei Tage dauernden Leitlinientreffen zusammen. Diese Kommission erhielt alle Evidenzprofile vor dem Treffen und stimmten dem angestrebten Prozess der Leitlinienentwicklung zu.

**Ergebnisse:** Es dauerte einen Monat, das Team zusammenzustellen und die Evidenzprofile zu entwickeln. Danach dauerte es nur etwa 5 Wochen, die Evidenzprofile zu überarbeiten und einen Leitlinienentwurf vor dem Treffen der Kommission auszuarbeiten. Ein Manuskriptentwurf wurde innerhalb von 10 Tagen nach dem Treffen fertiggestellt. Die Stärken dieses Prozesses beinhalten die Transparenz und die Geschwindigkeit, die für die Gestaltung dieser Leitlinien benötigt wurden. Der Prozess könnte durch eine Verringerung der Dauer, die benötigt wird, um Methodiker zur Erstellung der Evidenzprofile zu rekrutieren, verbessert werden. Weitere Entwicklungsarbeit ist nötig, um die Einbindung aller potentiell relevanten Gruppen zu gewährleisten und um den Nutzen der Leitlinien zu evaluieren.

**Interpretation:** Es ist möglich, evidenzbasierte Leitlinien systematisch und transparent in nur 2 Monaten zu entwickeln. Die Kosten für diese Arbeit sind aber für niedrig- und mitteleinkommensstarken Ländern zu hoch, um dies durchzuführen, und es wäre Verschwendung, wenn andere einkommensstarke Länder den Prozess nicht zusammen durchführen würden. WHO, und andere Organisationen, die systematische Ansätze für die Entwicklung von schnellen Leitlinien und Ratschlägen anwenden, können unterstützend tätig sein, indem sie transparente und robuste Prozesse auswählen, die die Adaptierung an spezielle Umstände erlaubt.
