# Supplementary material for: Transparent Development of the WHO Rapid Advice Guidelines
Source: PLoS Med. 2007 May 29;4(5):e119. doi: 10.1371/journal.pmed.0040119 (PMC1877972; doi:10.1371/journal.pmed.0040119)
Supplement: Alternative Language Abstract S2 — (27 KB DOC). [file pmed.0040119.sd003.doc]

Translation into Turkish by Dr. Bulen Ozbay

ÖZET

Amaç: Acil sağlık problemleri hızlı tavsiye gerektirir. Avian Influenza (H5N1 ) virus infeksiyonunun farmakolojik tedavisinde belirsizlikle karşılaşan üye ülkelerin gereksinimine cevap için hızlı tavsiye yönergeleri geliştirilmesi için sistematik ve şeffaf pilot testlerinin tanımını ve geliştirilmesini WHO adına yapmaktayız.

Metodlar: Mevsimsel influenzanın tedavisi ve önlenmesi ile ilgili randomize klinik çalışmaların mevcut sistematik taramalarındaki ve in vitro ve hayvan çalışmaları, olgu sunumları içeren az sayıda H5N1 infeksiyonu ile ilgili bulguları özetleyen tablolar hazırladık. İki günlük toplantıda influenza ile ilgili araştırıcılar ve metodolojistler H5N1 hastalarını tedavi eden deneyimli klinisyenler ve klinik eksperlerin bir araya geldiği panel gerçekleşti. Panel üyeleri toplantı öncesi verileri gözden geçirdi ve sonraki süreç hakkında fikir birliğine vardı.

Bulgular: Veri profillerini hazırlayan ekibin bir araya gelmesi bir ay aldı. Ekip bir araya geldikten sonra panel toplantısı ile ilgili kaba rehberlerin hazırlanması ve veri profillerinin gözden geçirilip hazırlanması 5 hafta sürdü. Panel toplantısından sonra 10 gün içinde yayın için kaba bir önyazı hazırlandı. Bu işlemin gücü WHO rehberlerinin hazırlanmasında geçen sürenin kısa olmasından ve şeffaflığından kaynaklanmaktadır. Bu süreç komisyonun veri profilleri için gereken zamanı kısaltarak daha çok pekiştirilebilir. Destekleyici kuruluş (stakeholder) katılımını kolaylaştırmak ve rehberin yararlılığını pekiştirmek, test etmek için rehberin daha fazla geliştirilmesine ihtiyaç vardır.

Yorum: İki ay gibi kısa süre içinde şeffaf ve sistematik olarak veri tabanlı rehberleri geliştirmek akılcıdır. Ancak, bunun maliyeti düşük ve orta gelir ülkeler için çok yüksek olmaktadır. Yüksek gelir grubu ülkeler için ise bu süreci gereksiz yere tekrarlamak israf olacaktır. WHO veya hızlı rehber geliştirmek için sistematik yaklaşım geliştiren diğer organizasyonlar tarafından özel durumlara uyumu basitleştiren güçlü ve şeffaf sürecin kullanılmasıyla bu önemli hizmet verilebilir.
