# Supplementary material for: Transparent Development of the WHO Rapid Advice Guidelines
Source: PLoS Med. 2007 May 29;4(5):e119. doi: 10.1371/journal.pmed.0040119 (PMC1877972; doi:10.1371/journal.pmed.0040119)
Supplement: Alternative Language Abstract S3 — (26 KB DOC). [file pmed.0040119.sd004.doc]

**Translation of abstract into Norwegian by Gunn E Vist**

**WHO rapid advise guideline: Raskt og etterprøvbart**

**Bakgrunn**: Nye helseproblemer kan kreve raske tiltak. Vi beskriver her utviklingen og pilottestingen av en systematisk og etterprøvbar metode brukt av verdens helseorganisasjon (WHO) til å utvikle en rapid advice guideline. Dette på oppfordring fra medlemsland som var usikre på beste farmakologiske behandling av sporadisk fugleinfluensa infeksjon (H5N1).

**Metode**: Som utgangspunkt for utvikling av rapid advice guideline lagde vi faktaprofiler. Disse summerte resultater fra systematiske oversikter av randomiserte kontrollerte studier om behandling og forebygging av sesongpreget influensa og tilgjengelig kunnskap om H5N1 infeksjon, inklusive pasient journaler, dyreforsøk og laboratorietester. Et panel av kliniske eksperter, klinikere med erfaring i å behandle pasienter med H5N1, influensaforskere og metodeforskere var samlet for et todagers møte. Panelmedlemmene vurderte dokumentasjonen på forhånd og godkjente prosessen.

**Funn**: Det tok en måned å sette sammen gruppen til å lage faktaprofilene. Da gruppen var klar tok det bare fem uker å lage og revidere faktaprofilene og å forberede et forslag til rapid advice guideline til panelmøtet. Et utkast til manuskript til publisering var klar ti dager etter panelmøtet. Fordelene ved denne fremgangsmåten er blant annet at den er transparent og den tar kort tid. Prosessen kan forbedres ved å redusere tiden som trengs for å bestille faktaprofilene. Videre utvikling trengs til å forenkle medvirkning av interessenter, og å forsikre og ivareta nytten av en rapid advice guideline.

**Tolkning**: Det er mulig å utvikle faktabaserte rapid advice guidelines systematisk og transparent på så kort tid som to måneder. Men kostnadene ved en slik prosess er uoverkommelige for lav og middels lavinntektsland og det ville være sløsing for de rike landene å duplisere denne prosessen unødvendig. WHO, og andre organisasjoner som følger systematiske fremgangsmetoder for å utvikle rapid advice guidelines, kan fremskaffe dette viktige tilbudet ved å bruke en robust og etterprøvbar prosess som forenkler tilpassning til spesifikke forhold.
