# Supplementary material for: Transparent Development of the WHO Rapid Advice Guidelines
Source: PLoS Med. 2007 May 29;4(5):e119. doi: 10.1371/journal.pmed.0040119 (PMC1877972; doi:10.1371/journal.pmed.0040119)
Supplement: Alternative Language Abstract S4 — (27 KB DOC). [file pmed.0040119.sd005.doc]

Translation of abstract into Italian by Dr. Irene Terrenato, Holger J. Schünemann and Prof. Paola Muti

***SOMMARIO***

Le malattie emergenti e le vaste problematiche ad esse connesse richiedono soluzioni immediate ed interventi tempestivi.

In relazione alla possibile diffusione della virus H5N1 (agente eziologico dell’influenza aviaria) l’Orgazizzazione Mondiale della Sanità (OMS) ha realizzato una specifica metodologia per lo sviluppo di linee guida che gli Stati Membri potranno adottare per affrontare gli aspetti terapeutici di questa condizione sanitaria di potenziale emergenza.

Questo sommario descrive le tappe fondamentali che hanno portato allo sviluppo delle linee guida adottate dall’OMS per i provvedimenti terapeutici in caso di diffusione di influenza aviaria

**Metodi:**

Inizialmente sono state preparate delle tabelle riassuntive delle revisioni sistematiche che sono state condotte per valutare l’evidenza prodotta da trial randomizzati riguardo all’efficacia di terapie relative al trattamento e alla prevenzione dell’influenza stagionale. E’ stata anche considerata l’evidenza non sperimentale disponibile includendo gli studi su specifici singoli casi di persone affette, gli studi condotti su animali e gli studi in vitro.

Esperti clinici con esperienza nel trattamento di pazienti affetti da H5N1, ricercatori specializzati nello studio di virus influenzali e metodologi clinici hanno valutato le evidenze preparate e concordato un criterio da seguire per sviluppare delle linee guida che tenessero conto della evidenza scientifica fin qui acquisita in questo campo.

**Risultati:**

In un mese sono stati preparati i profili delle evidenze e, nelle cinque settimane successive, sono stati revisionati per preparare una bozza di linee guida. Una volta discussa la bozza in un incontro con gli esperti, la prima stesura della versione finale è stata preparata nei successivi dieci giorni.

Lo sviluppo delle linee guida e’ stato quindi relativamente veloce e la discussione sulla evidenza e la forza delle evidenza analizzata e’ stato trasparente e partecipato. Il processo di sviluppo di linee guida potra’ in futuro essere migliorato riducendo il tempo necessario per definire la fase iniziale dei profili della evidenza e favorendo una migliore motivazione a partecipare da parte degli esperti clinici chiarendo loro il valore e l’utilita’ delle linee guida in campo sanitario..

**Interpretazione:**

E’ importante sviluppare delle linee guida basate sull’evidenza scientifica raccolta in campo sperimentale per offrire delle valide strategie diagnostiche terapeutiche al sistemi sanitari nazionali ed internazionali. Il processo di definizione di linee guida deve avvenire in maniera sistematica e trasparente e in un periodo relativamente breve (due mesi nel caso riportato). Tuttavia, il costo di questo processo e’ in genere assai elevato ed e’ problematico affrontarlo dai paesi a basso e medio reddito. Per questo motivo la OMS ed altre organizzazioni simili, interessate a seguire l’acquisizione sistematica dell’evidenza scientifica, può fornire questo importante servizio alla comunita’ internazionale utilizzando metodologie solide e chiare che permettano la fruizione dei prodotti del lavoro metodologico anche a realta’ diversificate di paesi culturalmente ed economicamente diversi.
