# Supplementary material for: Transparent Development of the WHO Rapid Advice Guidelines
Source: PLoS Med. 2007 May 29;4(5):e119. doi: 10.1371/journal.pmed.0040119 (PMC1877972; doi:10.1371/journal.pmed.0040119)
Supplement: Alternative Language Abstract S5 — (27 KB DOC). [file pmed.0040119.sd006.doc]

**Abstract translated into French by Dr. Yazdan Yazdanpanah**

**Introduction** : Les maladies émergentes nécessitent un partage rapide des informations et des connaissances. Ici, nous décrivons une approche systématique et transparente utilisée par l’OMS pour développer des recommandations rapides sur la prise en charge thérapeutique des infections au virus de la grippe aviaire A (H5N1). Ces recommandations ont été développées à la demande des états membres confortés à des incertitudes concernant la prise en charge de cette pathologie.

**Méthodes** : Nous avons tout d’abord préparé des documents résumant les résultats : (i) des revues systématiques des essais cliniques randomisés évaluant le traitement curatif et préventif de la grippe saisonnière ; (ii) des études disponibles sur le virus H5N1 et en particulier des cas cliniques et des études *in vitro*. Puis, un panel constitué par des experts cliniciens ayant déjà pris en charge des patients infectés par le virus de H5N1, des chercheurs dans le domaine de la grippe, et des méthodologistes a été réuni pour une durée de deux jours. Il a été demandé aux membres du panel d’étudier les documents résumant les résultats et les niveaux de preuves pour chaque indication thérapeutique avant la réunion.

**Résultats** : Le groupe de personnes qui a réalisé la revue systématique des données de la littérature a été constitué en quatre semaines. Ce groupe a revu sur une période de cinq semaines les données de la littérature sur le traitement de la grippe, a déterminé les niveaux de preuves pour chaque indication thérapeutique, et écrit une première version des recommandations avant la réunion du panel d’experts. Une première version d’un article a été préparé pour publication dans les dix jours suivant la réunion du panel d’experts. Les points forts de ce processus sont sa transparence et la rapidité avec la quelle il a permis de mettre en place des recommandations. Ce processus peut être toutefois amélioré en diminuant le temps nécessaire à constituer le groupe ayant réalisé la revue systématique des données et le choix des niveaux de preuves. Dans l’avenir, il faut améliorer l’implication des partenaires, et évaluer l’impact *a posteriori* de ces recommandations.

**Conclusion** : Il est possible de développer des recommandations en utilisant une approche systématique et transparente sur un laps de temps court. Toutefois, le coût de ce processus est élevé pour les pays à niveau de revenu bas ou moyen. Dans les pays à niveau de revenu élevé il est probablement inutile de dupliquer ce processus. L’OMS ou d’autres organisations, en suivant une approche systématique robuste et rapide peuvent développer ces recommandations qui pourraient être ensuite adaptées à des contextes spécifiques.
