# Supplementary material for: Transparent Development of the WHO Rapid Advice Guidelines
Source: PLoS Med. 2007 May 29;4(5):e119. doi: 10.1371/journal.pmed.0040119 (PMC1877972; doi:10.1371/journal.pmed.0040119)
Supplement: Alternative Language Abstract S6 — (26 KB DOC). [file pmed.0040119.sd007.doc]

**Translation into Chinese by Dr. Xiao-Ning Xu**

**摘要**

**背景：**解决新发传染病的问题需要快速的咨询。在此我们描述一个有发展潜力并具有指导性的实践手段。此方法具有系统性，且透明度高，是世界卫生组织根据各成员国的需要制定的，对禽流感病毒感染在不肯定的情况下药物治疗及管理的快速指南。

**方法：**我们利用表格的形式对目前季节性流感治疗与预防的临床双盲实验，及对人禽流感非临床实验所进行系统性回顾和总结。其中包括病例报告，动物实验以及体外实验的研究。召集一组具有临床经验，特别是对人禽流感治疗，且在流感病毒科研及试验方法有丰富经验的专家开一个两天的会议。于会前，专家组成员回顾了综合科研数据分析，并达成了程序上的共识。

**结果：**用了一个月的时间来组织专家组，并准备综合数据分析。一旦专家组成立，在开会前只需要五周的时间来准备综合数据分析的改写以及制定初步的应对指南。会议后10天内准备好发表指南的初稿。所述方法的强项在于高透明度，并能在很短时间内准备好世界卫生组织所需的指南。整个操作过程可以被进一步的改进，即通过缩短准备制定综合数据分析的时间。在投资或科研经费提供方的协助和参与下，此方法有必要进行进一步的发展，用以评估及确保指南的实用性。

**意义：**制定出以综合数据分析为基础的，系统性强及透明度高的指南是可行的。但是，这个操作程序所花费用，是主要阻止其在中低收入国家实施原因。而在生活高收入国家重复此程序是一个没有必要的浪费。世界卫生组织以及其它机构遵循系统性的手段来开发快速咨询，利用这样一个健全的，透明度高且简化的，适合当地国情的程序，以达到提供此重要服务的目的。
