# Supplementary material for: Transparent Development of the WHO Rapid Advice Guidelines
Source: PLoS Med. 2007 May 29;4(5):e119. doi: 10.1371/journal.pmed.0040119 (PMC1877972; doi:10.1371/journal.pmed.0040119)
Supplement: Alternative Language Abstract S7 — (28 KB DOC). [file pmed.0040119.sd008.doc]

**Translation of abstract into Pilipino (Tagalog) Dr Efren Dimaano:**

**Batayan:** Ang mga makabagong lumilitaw na problemang pangkalusugan ay nangangailangan ng mabilis na tagubilin. Ilalarawan natin ang pagusad at unang pagsubok ng maayos at malinaw na pamamaraang ginamit ng WHO upang makagawa ng panuntunan para matugunan ang mga pangangailangan ng mga bansang kasapi na hindi nakasisiguro sa mga gamot na ginagamit sa trangkasong Avian A(H5N1).

**Paraan:** Gumawa kami ng tabulasyon na nagbubuod ng mga kasalukuyang patukmo tukmong pananaliksik ukol sa gamutan at pag-iwas sa trangkasong H5N1, sa mga nakuhang ebidensyang di dumaan sa medotikong pagsisiyasat ng mga trangkasong H5N1, mga case report, sa mga pagsusuri ng mga hayop na naimpeksyon at mga pananaliksik sa mga laboratoryo. Isang lupon na binubuo ng mga experto sa paggagamot ng trangkasong H5N1, mga may karanasan sa paggagamot ng nasabing sakit mga mananaliksik at mga medotiko ay nagtipon at nagusap ng dalawang sunod na araw. Pinagaralan ng bawat miyembro ng lupon ang mga ebidensya bago sila magtipon.

**Resulta:** Inabot ng isang buwan ang pagbuo ng lupon na maghahanda ng perpil ng mga ebidensya. Pagkatapos, inabot naman ng limang linggo ang pghahanda at pagwawasto ng perpil ng mga ebidensya at pagbabalangkas ng panuntunan bago magusapusap ang lupon. Ang binalangkas na manuskrito para mailathala ay ginawa sampung araw makalipas ang pag-uusap. Ang tagumpay ng ganitong pamamaraan ay dahil sa malinaw at maigsing pag-uusap na ginugol para maihanda ang panuntunan ng WHO. Kailangan parin mahikayat ang pagsama ng mga nangangailangan at masuri at masiguro ang silbi ng panuntunan ng WHO.

**Interpretasyon:** Pwede pa lang makapagbalangkas ng maayos at malinaw na panuntunan na base sa mga ebidensiya sa loob lamang ng dalawang buwan. Lamang, ang paggawa ng ganito ay magastos para sa mga mahirap at sa medyo may kayang bansa at para naman sa mga bansang mayayaman na gustong gayahin ang ganitong pamamaraan ay di makabuluhan. Ang WHO o iba pang mga samahan na sumusunod sa ganitong pamamaraan upang makagawa ng mabilis na tagubilin ay makapagbibigay ng makabuluhang serbisyo sa pamamagitan ng paggamit ng malinaw at payak pamamaraan na angkop sa bawat sitwasyon.
