# Supplementary material for: Transparent Development of the WHO Rapid Advice Guidelines
Source: PLoS Med. 2007 May 29;4(5):e119. doi: 10.1371/journal.pmed.0040119 (PMC1877972; doi:10.1371/journal.pmed.0040119)
Supplement: Alternative Language Abstract S8 — (26 KB DOC). [file pmed.0040119.sd009.doc]

**Translation of abstract into Portugese by Martin Crusat**

**Sumário**

**Antecedentes:** Os problemas de saúde pública emergentes requerem um rápido assessoramento. Aqui descrevemos o desenvolvimento e ensaio piloto de um procedimento sistemático e transparente, utilizado pela OMS, para desenvolver as diretrizes de assessoramento rápido, necessárias em resposta às consultas dos estados membros que se enfrentem a dúvidas na gestão farmacológica de infecções causadas pelo vírus da gripe aviar (H5N1)

**Métodos:** Desenvolveram-se tabelas resumindo os achados publicados em revisões sistemáticas de ensaios clínicos aleatórios sobre tratamento e prevenção da gripe estacional, bem como as evidências não procedentes de ensaios clínicos sobre infecções com o vírus H5N1, incluindo casos publicados, estudos em modelos animais e ensaios in vitro. Um grupo de experientes, formado por pesquisadores e clínicos com experiência no tratamento de pacientes infectados com o vírus H5N1, reuniu-se durante um encontro de dois dias. Os membros deste comitê revisaram as evidências com anterioridade ao congresso e chegaram a um consenso no processo.

**Resultados:** Foi necessário um mês para reunir uma equipe capaz de preparar os perfis baseados nas evidências. Uma vez a equipe esteve formada foram necessárias somente cinco semanas para preparar e revisar estes perfis e preparar as diretrizes preliminares ao congresso. Dez dias depois do congresso esteve preparado para sua publicação um manuscrito preliminar. A importância deste processo radica em sua transparência e no escasso período de tempo empregado para preparar estas diretrizes da OMS. O processo poderia ser melhorado encurtando o tempo necessário para conformar os perfis baseados nas evidências. É necessário um maior desenvolvimento do processo para facilitar o compromisso dos interessados, bem como para avaliar e assegurar a utilidade da diretrizes.

**Interpretação:** É viável desenvolver diretrizes baseadas na evidência de uma forma sistemática e transparente num período de tempo tão breve como dois meses. No entanto o custo deste processo é prohibitivamente alto para países de meio e sob rendimento e séria um esbanje para os países de alto rendimento duplicar este processo desnecessariamente. A OMS, ou outras organizações que sigam um procedimento sistemático para o desenvolvimento de um assessoramento rápido, podem oferecer este serviço tão importante utilizando um processo robusto e transparente que simplifique a adaptação a situações específicos.

**Palavras finque:** diretrizes; saúde publica; doenças infecciosas; medicina baseada na evidência.
