# Supplementary material for: Transparent Development of the WHO Rapid Advice Guidelines
Source: PLoS Med. 2007 May 29;4(5):e119. doi: 10.1371/journal.pmed.0040119 (PMC1877972; doi:10.1371/journal.pmed.0040119)
Supplement: Alternative Language Abstract S9 — (27 KB DOC). [file pmed.0040119.sd010.doc]

**Translation of abstract into Spanish by Eric Martinez**

**RESUMEN**

**Antecedentes:** Los problemas emergentes de salud requieren rápida asesoria Nosotros describimos el desarrollo y prueba piloto de un enfoque sistemático y transparente utilizado por la OMS para desarrollar Guías de Asesoria en respuesta a solicitudes de los estados miembros que confronten dudas en el manejo farmacológico de la infección por el virus de la influenza aviar A(H5N1).

**Métodos:** Hemos preparado cuadros que resumen los resultados encontrados en las revisiones sistemáticas existentes de ensayos aleatorizados de tratamiento y prevención de la influenza estacional y la evidencia no obtenida mediante ensayos respecto a la infección por H5N1, incluyendo notificación de casos, estudios in vitro y en animales. Un panel integrado por expertos clínicos, clínicos con experiencia en el tratamiento de pacientes con H5N1, investigadores en influenza y metodologos fue convocado para una reunión de dos días. Los miembros del Panel revisaron la evidencia antes de la reunión y estuvieron de acuerdo con el proceso.

**Resultados:** Demoro un mes poder juntar el equipo para preparar los perfiles de evidencia. Una vez que el equipo estuvo constituido tomo solamente cinco semanas preparar y revisar los perfiles de evidencia y elaborar el borrador de las guías antes de la reunión del panel. El borrador de un manuscrito para publicación estuvo listo en 10 días después de la reunión del panel. Las fortalezas del proceso incluyen su transparencia y el corto tiempo requerido para preparar estas Guías de OMS. El proceso pudo haberse mejorado mediante el acortamiento del tiempo dedicado a los perfiles de evidencia. Se necesita un desarrollo ulterior para facilitar la participación de los decisores, evaluar y asegurar la utilidad de las Guías.

**Interpretacion:** Es factible desarrollar guías basadas en evidencia de forma sistemática y transparente en un periodo tan corto como dos meses. Sin embargo, el costo de hacer esto es prohibitivamente elevado para los países de ingresos bajos o medianos y seria un malgasto para los países de elevados ingresos duplicar este proceso innecesariamente..

La OMS u otras organizaciones que tienen enfoques sistemáticos para desarrollar asesoria rápida pueden ofrecer este importante servicio utilizando un proceso robusto y transparente que simplifica su adaptación a escenarios específicos.

**Palabras clave:** guías, salud pública, enfermedad infecciosa, medicina basada en evidencia
