# Supplementary material for: Transparent Development of the WHO Rapid Advice Guidelines
Source: PLoS Med. 2007 May 29;4(5):e119. doi: 10.1371/journal.pmed.0040119 (PMC1877972; doi:10.1371/journal.pmed.0040119)
Supplement: Alternative Language Abstract S10 — (30 KB DOC). [file pmed.0040119.sd011.doc]

***Translation of abstract into Indonesian by Dr Tjandra Yoga Aditama***

**Abstrak**

**Latar Belakang :** masalah kesehatan “*emerging*” memerlukan penanganan yang cepat. Kami menyampaikan perkembangan dan uji coba dari pendekatan sistematik dan transparan dari WHO untuk mewujudkan pedoman secara cepat sebagai respon dari negara anggota WHO sehubungan dengan belum terlalu jelasnya manajemen farmakologis infeksi virus Avian Influenza (H5N1)

**Metodologi :** Kami mempersiapkan tabel yang menggambarkan hasil pengamatan sistematik yang ada dari berbagai penelitian random tentang pengobatan dan pencegahan influenza biasa (bukan Flu Burung), dan data infeksi H5N1 lain seperti laporan kasus, penelitian binatang serta penelitian invitro. Kemudian data-data yang ada dianalisa oleh panel ahli dalam rapat selama 2 hari. Panel ahli ini terdiri dari ahli klinisi, klinisi yang punya pengalaman menangani pasien H5N1, peneliti influenza dan ahli metodologi penelitian. Para anggota panel ahli ini sudah mendapat dan menganalisa data-data yang ada sebelum rapat dimulai, dan mereka menyetujui proses yang terjadi

**Hasil :** Dibutuhkan waktu 1 bulan untuk membentuk team yang mempersiapkan bukti-bukti yang ada. Setelah team terbentuk, maka hanya diperlukan waktu 5 minggu untuk mempersiapkan dan merevisi bukti yang ada serta kemudian membuat draft petunjuk (*Guidelin*e) sebelum pertemuan panel ahli dilakukan. Draft manuskrip untuk publikasi dipersiapkan dalam waktu 10 hari setelah pertemuan dilakukan. Kekuatan proses ini meliputi adanya transparansi dan tidak panjangnya waktu yang dibutuhkan untuk membuat petunjuk (*Guidelin*e) WHO ini. Proses ini bahkan dapat diperbaiki dengan memperpendek waktu yang dibutuhkan untuk mengumpulkan bukti ilmiah yang ada. Diperlukan perkembangan selanjutnya dengan keterlibatan semua pihak yang terkait untuk mengevaluasi dan menilai manfaat petunjuk (*Guidelin*e) ini

*approaches for developing rapid advice, can provide this important service by using a robust and transparent process that simplifies adaptation to specific settings.*

**Interpertasi :** adalah layak untuk membuat petunjuk (*Guidelin*e) berbasis bukti secara sistematis dan trasparans dalam waktu 2 bulan saja. Tetapi, biaya untuk melakukannya adalah amat tinggi untuk dilakukan di negara berpenghasilan rendah dan menengah, dan nampaknya tidak berguna kalau negara berpenghasilan tinggi berupaya untuk menggandakan proses ini secara tidak perlu. WHO, atau badan lain yang mengikuti proses sistematik untuk memproduksi petunjuk secara cepat, dapat menggunakan pelayanan penting ini dengan cara yang baik dan transparan dengan menyederhanakan dan mengadaptasinya pada keadaan-keadaan khusus

**Kata Kunci :** petunjuk (*Guidelin*e) ; kesehatan masyarakat; penyakit infeksi; kedokteran berbasis bukti
