# Supplementary material for: Transparent Development of the WHO Rapid Advice Guidelines
Source: PLoS Med. 2007 May 29;4(5):e119. doi: 10.1371/journal.pmed.0040119 (PMC1877972; doi:10.1371/journal.pmed.0040119)
Supplement: Alternative Language Abstract S11 — (60 KB DOC). [file pmed.0040119.sd012.pdf]

Translation into Laos by Dr Mayfong Mayxay

ບົດຄັດຫຍໍ້

ຄວາມເປັນມາ: ບັນຫາສຸກເສີນທາງດ້ານສາທາລະນະສຸກ ແມ່ນຕ້ອງການຄຳແນະນຳໃນທັນທີທັນໃດ. ໃນທີ່ນີ້ ພວກເຮົາໄດ້ພັນລະນາກ່ຽວກັບ ການພັດທະນາ ແລະການທົດສອບ ແນວທາງ ຢ່າງເປັນຫລັກການ ແລະໂປ່ງໃສ (ທີ່ທາງອົງການອານາໄມໂລກເຄີຍໃຊ້ມາແລ້ວ) ເພື່ອເປັນບ່ອນອີງໃນການຂຽນເປັນປື້ມຄູ່ມື ສຳລັບໃຫ້ຄຳແນະນຳຢ່າງທັນການ ແນໃສ່ຕອບສະໜອງຕາມຄວາມຮຽກຮ້ອງຕ້ອງການຂອງບັນດາປະເທດສະມະຊິກ ທີ່ກຳລັງປະເຊີນໜ້າກັບຄວາມບໍ່ແນ່ນອນໃນເລື້ອງການປິ່ນປົວພະຍາດໄຂ້ຫວັດສັດປີກ ທີ່ເກີດຈາກການຕິດເຊື້ອ ໄວຣັສ H5N1.

ວິທີການສຶກສາຄົ້ນຄວ້າ: ພວກເຮົາໄດ້ທຳການທົບທວນຜົນຈາກການຄົ້ນຄວ້າແບບຂຸ່ມ ກ່ຽວກັບການປິ່ນປົວ ແລະປ້ອງກັນພະຍາດໄຂ້ຫວັດທີ່ເກີດຕາມລະດູການ ແລະ ທົບທວນຂໍ້ມູນຫລັກຖານທີ່ບໍ່ໄດ້ທຳການຄົ້ນຄວ້າໃນເລື້ອງການຕິດເຊື້ອໄວຣັສ H5N1, ລວມທັງໄດ້ທົບທວນບົດລາຍງານກໍລະນີຄົນເຈັບ, ການສຶກສາຄົ້ນຄວ້າ ໃນສັດ ແລະໃນຫລອດທົດລອງ ແລ້ວນຳມາສັງລວມໄວ້ເປັນຕາຕະລາງຈຳນວນໜື່ງ. ຈາກນັ້ນ ກໍ່ໄດ້ຈັດໃຫ້ມີການປະຊູມຂື້ນເປັນເວລາ 2 ວັນ ໂດຍໄດ້ເຊື້ອເຊີນເອົາຜູ້ຊ່ຽວຊານທາງດ້ານກລີນິກ, ແພດໝໍທີ່ມີປະສົບປະການໃນການປິ່ນປົວຄົນເຈັບຕິດເຊື້ອ H5N1, ນັກຄົ້ນຄວ້າກ່ຽວກັບພະຍາດໄຂ້ຫວັດ ແລະນັກວິໃຈທາງສະຖິຕິການແພດເຂົ້າຮ່ວມ. ກ່ອນການປະຊູມ, ຜູ້ເຂົ້າຮ່ວມປະຊູມໄດ້ທຳການທົບທວນຂໍ້ມູນຫລັກຖານທັງໝົດ ພ້ອມທັງເຫັນດີໃນເລື້ອງຂັ້ນຕອນຂອງການປະຊູມຢ່າງເປັນເອກະພາບກັນກ່ອນແລ້ວ.

ຜົນຂອງການສຶກສາ: ພວກເຮົາໄຊ້ເວລາ 1 ເດືອນ ໃນການຈັດຕັ້ງທີມງານເພື່ອເກັບກຳຂໍ້ມູນຫລັກຖານທັງໝົດ. ພາຍຫລັງທີ່ທີມງານຖືກແຕ່ງຕັ້ງຂື້ນ, ແມ່ນໄຊ້ເວລາພຽງແຕ່ 5 ອາທິດ ໃນການຈັດຫາ ແລະທົບທວນຂໍ້ມູນຫລັກຖານ ລວມທັງການຮ່າງປື້ມຄູ່ມື ຂື້ນ ກ່ອນທີ່ຈະຈັດໃຫ້ມີການປະຊູມຄະນະກຳມະການ. ຫລັງຈາກການປະຊູມຄະນະກຳມະການສີ້ນສຸດລົງ, ກໍ່ໄດ້ຮ່າງບົດທີ່ຈະຕຽມນຳໄປຕີພິມເຜີຍແຜ່ ຊື່ງໄຊ້ເວລາສຳລັບການກະກຽມພຽງ 10 ວັນ. ຈຸດດີຂອງຂັ້ນຕອນການຕຽມງານຄັ້ງນີ້ ແມ່ນໄດ້ແກ່ຄວາມໂປ່ງໃສ ແລະການໄຊ້ເວລາທີ່ສັ້ນ ສຳລັບຂຽນປື້ມຄູ່ມືຂອງອົງການອານາໄມໂລກເຫລັ້ມນີ້. ພວກເຮົາສາມາດເຮັດໃຫ້ຂັ້ນຕອນນີ້ດີຂື້ນຕື່ມ ດ້ວຍການເຮັດໃຫ້ເວລາທີ່ຕ້ອງການເຂົ້າໃນການຈັດຫາຂໍ້ມູນຫລັກຖານນັ້ນສັ້ນລົງຕື່ມ. ເຖີງຢ່າງໃດກໍ່ຕາມ, ຍັງມີຄວາມຈຳເປັນທີ່ຈະຕ້ອງເອື້ອອຳນວຍ ໃຫ້ມີການປະກອບສ່ວນຈາກຫລາຍຝ່າຍຕື່ມອີກ, ແລະຕ້ອງມີການປະເມີນ ແລະຮັບປະກັນໃຫ້ເຫັນວ່າ ປື້ມຄູ່ມືເຫລັ້ມດັ່ງກ່າວແມ່ນມີປະໂຫຍດຢ່າງແທ້ຈິງ.

ຄວາມໝາຍຂອງການສຶກສານີ້: ການສຶກສານີ້ຊີ້ໃຫ້ເຫັນວ່າ ມັນເປັນໄປໄດ້ທີ່ເຮົາຈະສ້າງຄູ່ມືສຳລັບໃຫ້ຄຳແນະນຳ ຈາກຂໍ້ມູນຫລັກຖານຕົວຈິງ ຢ່າງເປັນຫລັກການ ແລະໂປ່ງໃສໄດ້ ຊື່ງໄຊ້ເວລາສັ້ນໆພຽງ 2 ເດືອນ

ເທົ່ານັ້ນ. ເຖິງຢ່າງໃດກໍ່ຕາມ, ລາຄາໃນການສ້າງຄູ່ມືແບບນີ້ ແມ່ນຍັງຖືວ່າແພງເກີນໄປສຳລັບບັນດາປະເທດທີ່ມີລາຍຮັບໃນລະດັບຕ່ຳ ແລະປານກາງ ໃນຂະນະທີ່ມັນອາດເປັນການສິ້ນເປືອງໂດຍບໍ່ມີປະໂຫຍດຫຍັງສຳລັບບັນດາປະເທດທີ່ມີລາຍຮັບສູງ ຊຶ່ງຈະຕ້ອງມາເຮັດຕາມຂັ້ນຕອນແບບນີ້ໂດຍບໍ່ຈຳເປັນອີກ. ຈາກການນຳໃຊ້ແນວທາງສຳລັບພັດທະນາເຄື່ອງມືແບບໄວວາເຊັ່ນນີ້, ອົງການອານາໄມໂລກ ຫລືອົງການຈັດຕັ້ງອື່ນໆສາມາດທີ່ຈະໃຫ້ການບໍລິການທີ່ສຳຄັນໃນລັກສະນະເຊັ່ນນີ້ໄດ້ ດ້ວຍການນຳໃຊ້ຂັ້ນຕອນທີ່ເປັນຫລັກການ ແລະໂປ່ງໃສ ຊຶ່ງອາດສາມາດນຳໄປປັບປຸງໃຫ້ແທດເໝາະກັບສະພາບຕົວຈິງຂອງແຕ່ລະບ່ອນ ເພື່ອໃຫ້ມີລັກສະນະງ່າຍດາຍຂຶ້ນຕື່ມ.

ຂໍ້ຄວາມທີ່ສຳຄັນ: guidelines; public health; infectious disease; evidence based medicine
