# Supplementary material for: Transparent Development of the WHO Rapid Advice Guidelines
Source: PLoS Med. 2007 May 29;4(5):e119. doi: 10.1371/journal.pmed.0040119 (PMC1877972; doi:10.1371/journal.pmed.0040119)
Supplement: Alternative Language Abstract S12 — (34 KB DOC). [file pmed.0040119.sd013.doc]

**Translation of abstract into Vietnamese by Ms Tran Thi Hoang Chau and Dr Tuan**

**Baøi toùm löôïc**

**Ñaët vaán ñeà**: Caùc vaán ñeà y teá ñang troãi daäy thöôøng caàn ñi keøm vôùi caùc höôùng daãn ñieàu trò nhanh. Chuùng toâi moâ taû quaù trình phaùt trieån vaø vaän haønh thöû moät caùch thöùc tieáp caän coù heä thoáng vaø minh baïch ñaõ ñöôïc Toå Chöùc Y Teá Theá Giôùi(TCYT TG) aùp duïng trong vieäc xaây döïng caùc höôùng daãn ñieàu trò nhanh ñeå ñaùp öùng nhu caàu cuûa caùc quoác gia gaëp khoù khaên trong döï phoøng cuùm A veà maët döôïc laâm saøng.

**Phöông phaùp nghieân cöùu**: Chuùng toâi xaây döïng caùc baûng bieåu toång keát keát quaû caùc y vaên hoài cöùu coù tính heä thoáng hieän haønh veà thöû nghieäm laâm saøng ngaãu nhieân hoùa ñieàu trò vaø döï phoøng cuùm theo chu kyø muøa cuõng nhö caùc chöùng cöù coù giaù trò thaáp hôn, bao goàm caû caùc baùo caùo haøng loaït ca beänh, thöû nghieäm treân ñoäng vaät vaø trong phoøng thí nghieäm. Moät nhoùm chuyeân gia bao goàm caùc chuyeân gia laâm saøng, caùc baùc syõ coù kinh nghieäm ñieàu trò beänh nhaân cuùm H5N1, caùc nhaø nghieân cöùu cuùm cuøng caùc chuyeân gia veà maët phöông phaùp nghieân cöùu hình thaønh vaø thaûo luaän trong moät khoùa hoïp hai ngaøy lieân tieáp. Caùc thaønh vieân cuûa nhoùm chuyeân gia xem xeùt toaøn boä chuùng cöù hieän höõu tröôùc khoùa hoïp vaø ñoàng thuaän veà tieán trình cuûa caùc buoåi hoïp.

**Keát quaû**: Moät nhoùm thaønh vieân cuûa chuùng toâi thu thaäp caùc y vaên chöùng cöù trong voøng moät thaùng. Khi caùc chöùng cöù do caùc thaønh vieân ñöôïc hôïïp nhaát, chuùng toâi caàn theâm 5 tuaàn ñeå chuaån bò, cuûng coá döõ kieän vaø hoaøn taát baûn nhaùp höôùng daãn ñieàu trò ñeå göûi tôùi nhoùm caùc chuyeân gia. Baûn thaûo daønh rieâng cho coâng boá treân taïp chí chuyeân ngaønh ñöôïc thöïc hieän trong voøng 10 ngaøy sau khoùa hoïp. Öu ñieåm cuûa phöông thöùc tieáp caän naøy laø tính minh baïch vaø söû duïng quyõ thôøi gian ngaén ñeå xaây döïng caùc höôùng daãn ñieàu trò cho TCYT TG. Phöông thöùc tieáp caän naøy coù theå ñöôïc thöïc hieän nhanh hôn baèng caùch ruùt ngaén thôøi gian thu thaäp caùc chöùng cöù trong y vaên. Cuõng caàn caûi thieän tieáp tuïc phöông thöùc tieáp caän naøy ñeå ñaûm baûo moïi thaønh vieân ñaõ ñaùnh giaù vaø tham gia toaøn dieän cuõng nhö chaéc chaén veà khaû naêng höõu duïng cuûa höôùng daãn ñieàu trò ñaõ ñöôïc xaây döïng.

**Baøn luaän vaø keát luaän**: Coù theå xaây döïng caùc höôùng daãn ñieàu trò moät caùch heä thoáng vaø minh baïch döïa treân caùc chöùng cöù trong thôøi gian hai thaùng. Tuy vaäy, chi phí cuûa caùch tieáp caän naøy coøn töông ñoái cao ôû caùc quoác gia coù thu nhaäp bình quaân ñaàu ngöôøi thaáp hay trung bình vaø cuõng laõng phí cho caùc quoác gia coù thu nhaäp bình quaân ñaàu ngöôøi cao neáu laäp laïi caùc böôùc naøy moät caùch khoâng caàn thieát. TCYT TG vaø caùc toå chöùc khaùc coù söû duïng caùch tieáp caän heä thoáng ñeå xaây döïng nhanh caùc höôùng daãn ñieàu trò coù theå söû duïng phöông thöùc tieáp caän chaén chaén vaø minh baïch naøy ñeå aùp duïng theo ñieàu kieän ñaëïc thuø töøng quoác gia.

**Töø khoùa:** höôùng daãn ñieàu trò; y teá coäng coäng; beänh truyeàn nhieãm; y hoïc döïa treân chöùng cöù
