# Supplementary material for: Transparent Development of the WHO Rapid Advice Guidelines
Source: PLoS Med. 2007 May 29;4(5):e119. doi: 10.1371/journal.pmed.0040119 (PMC1877972; doi:10.1371/journal.pmed.0040119)
Supplement: Alternative Language Abstract S13 — (26 KB DOC). [file pmed.0040119.sd014.doc]

**Translation of abstract into Finish by Heikki Peltola**

**LYHENNELMÄ (abstrakti)**

**Tausta:**. Uudet terveysongelmat edellyttävät nopeaa reagointia. Me kuvaamme kokeellisen, mutta jo WHO:nkin hyödyntämän järjestelmän, jossa jäsenvaltioita ohjeistetaan ("guide lines"), kuinka toimia lintuinfluenssan (H5N1) farmakologisen hoidon suhteen.

**Metodit:** Me taulukoimme tiedot, jotka saatiin satunnaistetuista tavanomaista influenssaa koskevista hoito- ja estotutkimuksista, H5N1-tartunnasta kertyneestä informaatiosta, yksittäisistä potilasraporteista sekä eläin- ja in vitro -tutkimuksista. Kaikki tieto käsiteltiin kaksipäiväisessä paneelissa, jossa oli edustettuna mahdollisimman edustava joukko H5N1- ja muita influenssa-asiantuntijoita.

**Havainnot:**  Kuukauden kestäneen valmistelujakson jälkeen kului vain viisi viikkoa tuottaa tekstiluonnos paneelin käsiteltäväksi. Alustava käsikirjoitus syntyi 10 päivässä paneelin jälkeen. Prosessin etuja oli sen läpinäkyvyys ja jokseenkin lyhyt aika, joka vaadittiin näinkin laajan WHO:n ohjeistuksen synnyttämiseksi. Prosessia voisi parantaa lyhentämällä valtuuksien saamista sekä helpottamalla osakkaiden aktiivia panostusta.

**Tulkinta:**  Ohjeistuksen saa laadituksi niinkin lyhyessä ajassa kuin kahdessa kuukaudessa, mutta se vaatii varoja eikä onnistune köyhissä tai keskituloisissa maissa. Toisaalta ei varakkaidenkaan maiden pidä vain tarpeettomasti toistaa koko prosessia. WHO tai jokin muu järjestelmällisesti toimiva organisaatio voi tuottaa myös tämän tärkeän palvelun, jos se turvautuu realiteetit huomioivaan, läpinäkyvään toimintaan. Tuollaista ohjeistusta voi sitten soveltaa tarpeen mukaan erilaisiin oloihin.

AVAINSANAT. Ohjeet, ohjeistus, guide lines, tartuntataudit, infektiot, näyttöön perustuva lääketiede, evidence based medicine.
