# Supplementary material for: Transparent Development of the WHO Rapid Advice Guidelines
Source: PLoS Med. 2007 May 29;4(5):e119. doi: 10.1371/journal.pmed.0040119 (PMC1877972; doi:10.1371/journal.pmed.0040119)
Supplement: Alternate Language Abstract S14 — (76 KB PDF). [file pmed.0040119.sd015.pdf]

요약문

배경: 최근에 발생한 보건 문제들은 신속한 권고를 필요로 한다. 우리는 조류독감 A (H5N1)형 바이러스 감염의 약리적 관리에 대한 불확실성에 직면한 회원국들의 요청에 부응하여 신속한 권고 지침을 개발하기 위해 WHO 가 사용하는 체계적이고 투명한 접근법의 개발과 실험테스트를 설명하였다

방법: 계절적 독감의 치료와 예방에 대한 무작위 표본 추출 임상 연구와 H5N1 감염에 대한 증례 보고, 동물 및 체외 실험 연구를 포함하는 관련 문헌에 대한 체계적 연구 내용을 요약한 표들을 준비하였다. 임상전문가, H5N1 환자 치료 경험이 있는 임상의, 인플루엔자 연구원 및 방법론가들로 구성된 패널들이 이틀간의 단일 미팅에 소집되었다. 패널 참석자들은 미팅 이전에 근거를 검토하고 이 과정에 동의하였다.

발견: 한 팀을 이뤄 근거 자료집을 준비하는 데 한 달이 소요되었다. 일단 팀이 결성된 후 패널 미팅에 앞서 근거 자료집을 준비하고 교정과 지침 초안을 준비하는 데는 단지 5 주가 소요되었다. 패널 미팅 후 10 일 이내에 출간을 위한 원고 초안이 준비되었다. 이 과정의 강점으로 투명성과 WHO 지침 마련을 위한 짧은 준비 소요 시간을 들 수 있는데, 이 과정은 근거 자료집을 의뢰하는 데 소요되는 시간을 단축함으로써 향상될 수 있다. 이해관계자의 참여를 용이하게 하고, 지침의 유용성을 평가하고 확보하는 데에 보다 개발이 필요하다.

해석: 2 개월 만에 체계적이고 투명하게 근거 중심의 지침을 개발하는 것은 가능하다. 그러나, 중간 및 저소득 국가들이 실행하기에는 비용이 엄청나게 높고 고소득 국가들이 이러한 과정을 불필요하게 중복하는 것은 낭비이다. 신속한 권고를 개발하기 위한 체계적 접근법에 따라 WHO 또는 다른 조직이,특정 환경에의 적용을 용이하게 하는 확실하고 투명한 과정을 사용함으로서 이러한 주요한 서비스를 제공할 수 있다

키워드: 지침, 보건, 전염병, 근거 중심 의학
