# Supplementary material for: Transparent Development of the WHO Rapid Advice Guidelines
Source: PLoS Med. 2007 May 29;4(5):e119. doi: 10.1371/journal.pmed.0040119 (PMC1877972; doi:10.1371/journal.pmed.0040119)
Supplement: Alternate Language Abstract S15 — (112 KB PDF). [file pmed.0040119.sd016.pdf]

背景：突発する保健衛生問題には、迅速なアドバイスが必要である。WHO の加盟国の中には鳥インフルエンザＡ型（H5N1）ウイルス感染における薬剤治療法が確立されていないために困惑した国もある。そういった国からの要請に応じて、WHO で使われているアドバイス・ガイドラインを迅速に制定するための順序だてられた透明性のあるアプローチの構築とそのパイロットテストを解説する。

方法：通常のインフルエンザの治療と予防のランダム化試験結果と、H5N1 感染における症例報告、動物実験結果、インビトロ実験結果をまとめて表記した。臨床専門医、H5N1 患者の治療経験のある臨床医、インフルエンザの研究従事者と方法論学者からなる委員会で 2 日間の会議を行った。委員会のメンバーは前述の科学的根拠の表を事前に検討し、プロセスについて同意した。

結果：科学的根拠をまとめるためのチームを発足させることに 1 ヵ月を要した。チームの発足後、科学的根拠のプロフィールの修正、草案ガイドラインの作成を委員会会合前に 5 週間をかけて準備した。出版のための草案原稿は、委員会会合の後、10 日以内に用意された。
この方法によって透明性のある WHO ガイドラインを、短期間に準備することができた。科学的根拠を取りまとめる期間をさらに迅速にできれば、全体にかかる時間を今以上に短縮できる。更には、関係者の参加を容易にすることと、ガイドラインの有用性の評価が必要である。

解釈：順序だてられた、透明性のある、科学的根拠に基づいたガイドラインを 2 ヵ月程度で制定することが可能である。しかし、このプロセスは低・中所得国には非常にコストがかかる。さらには、高額所得国が必要以上にこのプロセスを繰り返すことも浪費的である。
WHO、もしくはこのようなアドバイスのできる他の組織が、強力で透明性のあるプロセスを用いて、特定の国・地域への適合を容易にできるガイドラインの制定といった形でのサービスを提供するべきである。
