# Supplementary material for: Transparent Development of the WHO Rapid Advice Guidelines
Source: PLoS Med. 2007 May 29;4(5):e119. doi: 10.1371/journal.pmed.0040119 (PMC1877972; doi:10.1371/journal.pmed.0040119)
Supplement: Alternate Language Abstract S16 — (117 KB PDF). [file pmed.0040119.sd017.pdf]

__สำเนาคัดย่อ__

ภูมิหลัง: ปัญหาสุขภาพจำเป็นต้องมีการให้คำปรึกษาที่รวดเร็ว เราได้อธิบายพัฒนาการและการทดสอบนำร่องวิธีการที่เป็นระบบและโปร่งใสที่ใช้โดยองค์การอนามัยโลก ในการพัฒนาแนวทางการให้คำปรึกษาที่รวดเร็ว ที่เป็นการตอบสนองคำร้องขอจากประเทศสมาชิก ที่เผชิญกับความไม่แน่นอนเกี่ยวกับการจัดการด้านเภสัชวิทยาเกี่ยวกับการติดเชื้อไวรัส โรคไข้หวัดนก

วิธีการ: เราได้จัดทำตารางการทดสอบการตรวจทานที่เป็นระบบ เกี่ยวกับการสุ่มทดลองในการบำบัดรักษาและป้องกันโรคไข้หวัดนก และหลักฐานที่มิใช่จากการทดลองเกี่ยวกับการติดเชื้อ H5N1 รวมทั้งการรายงานเหตุการณ์ศึกษาในสัตว์ และการศึกษาในหลอดแก้วทีมงานที่ประกอบด้วยผู้ชำนาญทางการแพทย์ แพทย์คลินิกที่มีประสบการณ์ในการรักษาคนไข้ H5N1 นักวิจัยโรคไข้หวัดใหญ่ และผู้ศึกษาเกี่ยวกับกฎเกณฑ์ถูกจัดตั้งขึ้นสำหรับการประชุมเป็นเวลาสองวัน คณะทีมงานจะตรวจสอบหลักฐานก่อนที่จะมีการประชุม และได้ตกลงเกี่ยวกับกระบวนการ

การตรวจสอบ: ใช้เวลาหนึ่งเดือนในการรวบรวมทีมงานเพื่อเตรียมรายการหลักฐาน เมื่อมีการรวบรวมทีมงานใช้เวลาเพียงห้าสัปดาห์ในการเตรียม และปรับปรุงแก้ไขรายการหลักฐาน และเตรียมร่างแนวทางก่อนที่จะมีการประชุมทีมงาน มีการทำร่างต้นฉบับเพื่อตีพิมพ์เผยแพร่ภายใน 10 วัน หลังจากมีการประชุมทีมงาน ความแข็งแรงของกระบวนการจะรวมทั้งความโปร่งใส และระยะเวลาที่สั้นที่ใช้ในการเตรียมแนวทางขององค์การอนามัยโลก อาจมีการปรับปรุงกระบวนการโดยลดระยะเวลาที่ต้องใช้ในการทำรายการหลักฐาน นอกจากนี้จำเป็นต้องมีการพัฒนาเพื่อให้สะดวกต่อการถือหุ้น เพื่อประเมินผล และรับประกันความเป็นประโยชน์ของแนวทาง

การตีความ: มีความเป็นไปได้ที่จะพัฒนาแนวทางตามหลักฐาน อย่างเป็นระบบและโปร่งใสภายในเวลา สองเดือน อย่างไรก็ตามค่าใช้จ่ายในการดำเนินการค่อนข้างสูงสำหรับประเทศที่มีเงินได้ต่ำและปานกลาง และจะเป็นการเสียเวลาสำหรับประเทศที่มีรายได้สูงที่จะจำลองกระบวนการนี้โดยไม่จำเป็น องค์การอนามัยโลก หรือ องค์การอื่นๆ ที่ใช้วิธีการที่เป็นระบบในการพัฒนาการให้คำปรึกษาที่รวดเร็ว สามารถให้คำปรึกษาที่สำคัญนี้ โดยใช้กระบวนการที่แข็งแรงและโปร่งใสที่จะทำให้ง่ายต่อการปรับตัวเข้ากับสภาวะต่างๆ

คำที่สำคัญ: แนวทาง,สาธารณสุข,โรคติดต่อ,การแพทย์ตามหลักฐาน
