# Supplementary material for: Transparent Development of the WHO Rapid Advice Guidelines
Source: PLoS Med. 2007 May 29;4(5):e119. doi: 10.1371/journal.pmed.0040119 (PMC1877972; doi:10.1371/journal.pmed.0040119)
Supplement: Alternate Language Abstract S17 — (42 KB PDF). [file pmed.0040119.sd018.pdf]

भूमिका:

समय–समयमा देखा परिरहने नयाँ स्वास्थ्य समस्याहरुबारे यथाशीघ्र उचित निर्देशनहरु आवश्यक पर्दछन् । बर्डफ्लु (H5N1 Influenza) रोगको औषधोपचारबारे अनभिज्ञताको समस्या समाना गरेका सदस्य राष्ट्रहरुका अनुरोध बमोजिम विश्व स्वास्थ्य संगठनले निर्देशिका तयार पारेको थियो । सोही निर्देशिका बनाउन प्रयोग गरिएको विधिवत र पारदर्शी प्रणालीको यहाँ वर्णन गरिएको छ ।

विधि :

शुरुमा संक्षिप्त तालिकाहरु बनाइयो । ती तालिकाहरुमा मौसमी ज्वरो (Seasonal Influenza) को उपचार र रोकथामको अध्ययन गर्न हालसम्म गरिएका समानुपातिक अध्ययन–परिक्षण (Randomized Trials) का विधिवत समीक्षाहरु राखियो । यसका साथै वर्डफ्लु रोगको अध्ययन–परिक्षण नगरिएका प्रमाणहरु (जस्तैः चिकित्साधीन रोगीको विवरण, जनावरमा गरिएका अध्ययन र कृत्रिम अवस्थामा गरिएका अध्ययन) पनि ती तालिकामा समावेश गरियो । त्यसपछि चिकित्साविद्, वर्डफ्लू रोगको उपचारमा अनुभवी डाक्टरहरु र मौसमी ज्वरोको अनुसन्धानमा संलग्न व्यक्तिहरु सहितको एक समिति गठन गरियो । सो समितिले एक दुई–दिन–लामो सभा गर्‍यो । त्यस पूर्व नै समितिका सदस्यहरुले प्रमाणहरुको अध्ययन गरेर तरिकामा सहमत भइसकेका थिए ।

नतिजा:

विभिन्न अध्ययन र प्रमाणहरुको रुपरेखा तयार पार्ने टोली गठन गर्न मात्र ३० दिन लाग्यो । त्यति गरेपछि ३५ दिनभित्रै ती रुपरेखाको तालिका तयार भयो र संशोधन समेत पुरा भइ एक निर्देशिकाको प्रारुप तयार भयो र सो प्रारुप समितिको सभामा प्रस्तुत गरियो । सभाको १० दिनमा प्रकाशनको निम्ति एउटा निर्देशिकाको पाण्डुलिपीको प्रारुप तयार भयो । यो विश्व स्वास्थ्य संगठनको निर्देशिका तयार पार्न प्रयोग गरिएको प्रक्रिया पारदर्शी हुनु र छोटो समयमै पूरा हुन यस प्रक्रियाका गुणहरु हुन् । प्रमाणहरुको रुपरेखा तयार गर्ने टोलीलाई खटाउन लाग्ने समयलाई छोट्याएर यो प्रक्रियालाई अझ सुधार्न सकिन्छ । सबै सम्वन्धित पक्षका संलग्नतालाई अनुकुल पार्न र निर्देशिकाको उपयोगिता मुल्याङ्कन र सुनिश्चित गर्न थप कार्य आवश्यक पर्नेछन् ।

निष्कर्ष :

प्रमाणमा आधारित निर्देशिका विधिवत र पारदर्शी ढङ्गले दुई महिना भित्रै तयार गर्न सम्भव हुन्छ । तथापि, यो काम मध्य र निम्न आयवर्गका राष्ट्रहरुलाई अत्यन्तै खर्चिलो हुन्छ भने उच्च आयवर्गकाहरुलाई यो काम त्यसै दोहोर्‍याएर गर्नुपर्दा अपव्ययी हुन्छ । विभिन्न परिस्थितिमा प्रयोग गर्न अनुकुल, दरिलो र पारदर्शी प्रक्रिया प्रयोग गरेर विश्व स्वास्थ्य संगठन वा अरु संगठनहरु (जुन यथाशीघ्र निर्देशिका तयार पार्न विधिवत प्रणाली प्रयोग गर्छन) ले महत्वपूर्ण सेवा प्रदान गर्न सक्छन् ।

मुख्य शब्दहरु : निर्देशिका, जनस्वास्थ्य , संक्रामक रोग, प्रमाणमा आधारित चिकित्सा पद्दति
